# Multiple system atrophy with amyloid-β-predominant Alzheimer’s disease neuropathologic change

**DOI:** 10.1093/braincomms/fcae141

**Published:** 2024-04-17

**Authors:** Tomoya Kon, Shojiro Ichimata, Daniel G Di Luca, Ivan Martinez-Valbuena, Ain Kim, Koji Yoshida, Abdullah A Alruwaita, Galit Kleiner, Antonio P Strafella, Shelley L Forrest, Christine Sato, Ekaterina Rogaeva, Susan H Fox, Anthony E Lang, Gabor G Kovacs

**Affiliations:** Tanz Centre for Research in Neurodegenerative Disease, University of Toronto, Toronto, ON M5T 0S8, Canada; Department of Neurology, Hirosaki University Graduate School of Medicine, Hirosaki 036-8562, Japan; Tanz Centre for Research in Neurodegenerative Disease, University of Toronto, Toronto, ON M5T 0S8, Canada; Department of Legal Medicine, Faculty of Medicine, University of Toyama, Toyama 930-0194, Japan; Division of Neurology, Department of Medicine, University of Toronto, Toronto, ON M5S 1A1, Canada; Department of Neurology, Washington University in St. Louis, St. Louis, MO 63110, USA; Tanz Centre for Research in Neurodegenerative Disease, University of Toronto, Toronto, ON M5T 0S8, Canada; Tanz Centre for Research in Neurodegenerative Disease, University of Toronto, Toronto, ON M5T 0S8, Canada; Tanz Centre for Research in Neurodegenerative Disease, University of Toronto, Toronto, ON M5T 0S8, Canada; Department of Legal Medicine, Faculty of Medicine, University of Toyama, Toyama 930-0194, Japan; Edmund J Safra Program in Parkinson’s Disease and Rossy Program in Progressive Supranuclear Palsy, Toronto Western Hospital, Toronto, ON M5T 2S8, Canada; Neurology Department, Prince Sultan Military Medical City, Riyadh 11159, Saudi Arabia; Division of Neurology, Department of Medicine, University of Toronto, Toronto, ON M5S 1A1, Canada; Movement Disorders and Spasticity Management Clinic, Pamela and Paul Austin Centre for Neurology and Behavioral Support, Baycrest Centre for Geriatric Care, Toronto, ON M6A 2E1, Canada; Division of Neurology, Department of Medicine, University of Toronto, Toronto, ON M5S 1A1, Canada; Edmund J Safra Program in Parkinson’s Disease and Rossy Program in Progressive Supranuclear Palsy, Toronto Western Hospital, Toronto, ON M5T 2S8, Canada; Tanz Centre for Research in Neurodegenerative Disease, University of Toronto, Toronto, ON M5T 0S8, Canada; Laboratory Medicine Program & Krembil Brain Institute, University Health Network, Toronto, ON M5T 0S8, Canada; Faculty of Medicine, Health and Human Sciences, Dementia Research Centre, Macquarie Medical School, Macquarie University, Sydney, NSW 2109, Australia; Tanz Centre for Research in Neurodegenerative Disease, University of Toronto, Toronto, ON M5T 0S8, Canada; Tanz Centre for Research in Neurodegenerative Disease, University of Toronto, Toronto, ON M5T 0S8, Canada; Edmund J Safra Program in Parkinson’s Disease and Rossy Program in Progressive Supranuclear Palsy, Toronto Western Hospital, Toronto, ON M5T 2S8, Canada; Tanz Centre for Research in Neurodegenerative Disease, University of Toronto, Toronto, ON M5T 0S8, Canada; Division of Neurology, Department of Medicine, University of Toronto, Toronto, ON M5S 1A1, Canada; Edmund J Safra Program in Parkinson’s Disease and Rossy Program in Progressive Supranuclear Palsy, Toronto Western Hospital, Toronto, ON M5T 2S8, Canada; Tanz Centre for Research in Neurodegenerative Disease, University of Toronto, Toronto, ON M5T 0S8, Canada; Division of Neurology, Department of Medicine, University of Toronto, Toronto, ON M5S 1A1, Canada; Edmund J Safra Program in Parkinson’s Disease and Rossy Program in Progressive Supranuclear Palsy, Toronto Western Hospital, Toronto, ON M5T 2S8, Canada; Laboratory Medicine Program & Krembil Brain Institute, University Health Network, Toronto, ON M5T 0S8, Canada; Faculty of Medicine, Health and Human Sciences, Dementia Research Centre, Macquarie Medical School, Macquarie University, Sydney, NSW 2109, Australia; Department of Laboratory Medicine and Pathobiology, University of Toronto, Toronto, ON M5S 1A8, Canada

**Keywords:** alpha-synuclein, Alzheimer’s disease, amyloid-β, multiple system atrophy, tau

## Abstract

Multiple system atrophy is a neurodegenerative disease with α-synuclein pathology predominating in the striatonigral and olivopontocerebellar systems. Mixed pathologies are considered to be of low frequency and mostly comprise primary age-related tauopathy or low levels of Alzheimer’s disease-related neuropathologic change. Therefore, the concomitant presence of different misfolded proteins in the same brain region is less likely in multiple system atrophy. During the neuropathological evaluation of 21 consecutive multiple system atrophy cases, we identified four cases exhibiting an unusual discrepancy between high Thal amyloid-β phase and low transentorhinal Braak neurofibrillary tangle stage. We mapped α-synuclein pathology, measured the size and number of glial cytoplasmic inclusions and compared the amyloid-β peptides between multiple system atrophy and Alzheimer’s disease. In addition, we performed α-synuclein seeding assay from the affected putamen samples. We performed genetic testing for *APOE*, *MAPT*, *PSEN1*, *PSEN2* and *APP*. We refer to the four multiple system atrophy cases with discrepancy between amyloid-β and tau pathology as ‘amyloid-β-predominant Alzheimer’s disease neuropathologic change-multiple system atrophy’ to distinguish these from multiple system atrophy with primary age-related tauopathy or multiple system atrophy with typical Alzheimer’s disease neuropathologic change. As most multiple system atrophy cases with mixed pathologies reported in the literature, these cases did not show a peculiar clinical or MRI profile. Three amyloid-β-predominant Alzheimer’s disease neuropathologic change-multiple system atrophy cases were available for genetic testing, and all carried the *APOE* ɛ4 allele. The extent and severity of neuronal loss and α-synuclein pathology were not different compared with typical multiple system atrophy cases. Analysis of amyloid-β peptides revealed more premature amyloid-β plaques in amyloid-β-predominant Alzheimer’s disease neuropathologic change-multiple system atrophy compared with Alzheimer’s disease. α-Synuclein seeding amplification assay showed differences in the kinetics in two cases. This study highlights a rare mixed pathology variant of multiple system atrophy in which there is an anatomical meeting point of amyloid-β and α-synuclein, i.e. the striatum or cerebellum. Since biomarkers are entering clinical practice, these cases will be recognized, and the clinicians have to be informed that the prognosis is not necessarily different than in pure multiple system atrophy cases but that the effect of potential α-synuclein-based therapies might be influenced by the co-presence of amyloid-β in regions where α-synuclein also aggregates. We propose that mixed pathologies should be interpreted not only based on differences in the clinical phenotype but also on whether protein depositions regionally overlap, potentially leading to a different response to α-synuclein-targeted therapies.

## Teaser

Kon *et al*. report multiple system atrophy cases with a discrepancy between high Thal amyloid-β phase and low Braak tangle stage lacking specific clinical features but showing anatomic meeting points for amyloid-β and α-synuclein. They propose that mixed pathologies should be interpreted also based on regional overlap of proteins.

## Graphical Abstract

**Graphical Abstract:**
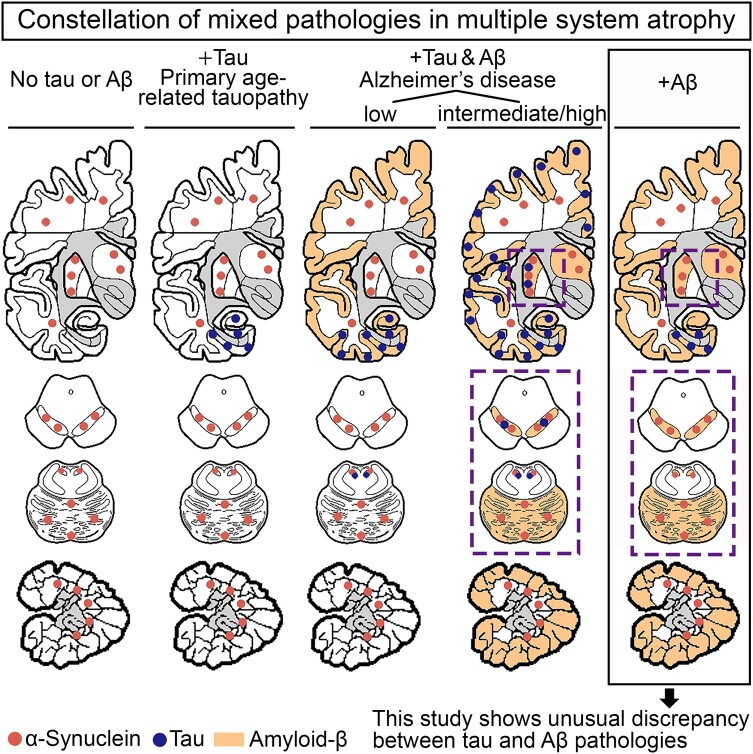

## Introduction

Multiple system atrophy (MSA) is a progressive neurodegenerative disease characterized by various combinations of parkinsonism, cerebellar ataxia and autonomic dysfunction.^1,2^ Pathologically, MSA is associated with predominant neuronal loss in the striatonigral and/or olivopontocerebellar systems, accompanied by the presence of characteristic α-synuclein (α-syn)-immunoreactive (ir) glial cytoplasmic inclusions (GCIs) distributed throughout the central nervous system.^1,2^

Mixed pathologies are frequently observed in neurodegenerative diseases, which contribute to clinical symptoms.^3-6^ Among these, Alzheimer’s disease-related neuropathologic change (ADNC) is the most frequent, characterized by the concomitant presence of neurofibrillary tangles (NFTs) and amyloid-β (Aβ) plaques both following a characteristic hierarchical sequence.^7-9^ In typical ADNC, the six stages of NFT and the five phases of Aβ pathologies increase in parallel. Cases with mild to moderate NFT pathology (Braak NFT Stages I–IV) and an absence of or minimal Aβ plaques (Thal Phases 0–2) are referred to as primary age-related tauopathy (PART).^10^ On the contrary, extensive Aβ pathology involving subcortical regions, the brainstem and cerebellum (i.e. Thal Phase 4 or 5) with tau pathology restricted to the transentorhinal/entorhinal regions (i.e. Braak Stage I or II) is a rare phenomenon: in a recent large international cohort, it represented 41 out of 2334 (1.7%).^11^ Although mixed pathologies are usually mild, they can be observed in MSA patients^5,6,12-17^ and typically comprise PART or low-level ADNC.

In this study, we describe four cases of MSA with a notable mixed pathology, revealing a remarkable discrepancy of widespread, high Thal phase and severe Aβ but minimal, low Braak stage tau pathology. We raise awareness of this variant, particularly since it provides the opportunity for the interaction of Aβ and α-syn in MSA-strategic anatomical regions, carrying the potential to influence the response to α-syn-targeted therapies. This supports the notion that cases with mixed proteinopathies might relate to distinct ‘strain-like’ features compared with those accumulating only one protein.^5,6,17^

## Materials and methods

### Case materials

We included 21 consecutive MSA and 124 consecutive non-MSA cases from the University Health Network Neurodegenerative Brain Collection, University of Toronto (Toronto, Canada) between 1982 and 2023. For the non-MSA cases, we included cases where the apolipoprotein E (*APOE*) genotype was available (for details, see Supplementary Table 1). Clinical details were obtained from a retrospective review of clinical records. All brains were obtained post-mortem through appropriate consenting procedures with Local Ethical Committee approval. This study received approval from the University Health Network Research Ethics Board (Nr. 20-5258) and the University of Toronto (Nr. 39459), adhering to the ethical standards established in the 1964 Declaration of Helsinki, updated in 2008.

### Neuropathologic assessment

Routine histological examination and immunohistochemistry were performed on 4-μm formalin-fixed paraffin-embedded tissue sections, targeting different Aβ epitopes,^18,19^ including pan-Aβ (6F/3D), Aβ_40_, Aβ_42_, Aβ_43_, pyroglutamate Aβ at the third glutamic acid (Aβ_Np3E_), phosphorylated-Aβ at the eighth serine (Aβ_pSer8_), anti-phosphorylated-tau, anti-α-syn and anti-phosphorylated TDP-43 antibodies. Supplementary Table 2 summarizes the antibodies and immunostaining pre-treatments used in this study. Immunostaining was conducted using the Dako Autostainer Link 48 and EnVision FLEX+ Visualization System, following the manufacturer’s instructions. All sections were subsequently counterstained with haematoxylin.

ADNC was assessed following the National Institute on Aging-Alzheimer’s Association guidelines.^9^ Additionally, the types of cerebral amyloid angiopathy (CAA),^20^ Lewy,^21^ TDP-43^22^ and vascular pathologies^21,22^ including arteriolosclerosis, infarcts and haemorrhages were evaluated.

In addition to the staging system described above, we semi-quantitatively graded the severity of neuronal loss, α-syn-ir neuronal cytoplasmic inclusions (NCIs) and GCIs and Aβ plaques using a 5-point scoring system as follows: Score 0, absence of pathology; Score 1, minimal; Score 2, mild; Score 3, moderate; and Score 4, severe.^23,24^ Neuronal loss and vascular lesions were assessed on haematoxylin and eosin (H&E) staining. The severity of CAA was scored under a 10× objective lens as follows: Score 0, no CAA; Score 1, occasional blood vessels with CAA (<20%); Score 2, a moderate number (20–60%) of blood vessels with CAA; and Score 3, many (>60%) blood vessels with CAA.^23,25^

### Morphometry of α-syn-ir GCIs

The morphological variables (i.e. the size, number and area density of all α-syn pathology) in the putamen and cerebellum were quantified following previously established protocols^26^ in four cases of Aβ-predominant ADNC-MSA (MSA 1–4) and 12 cases of non-Aβ-predominant ADNC-MSA (MSA 6, 8–12 and 15–20). The details of the method are provided in the Supplementary method. In brief, sections of the basal ganglia and cerebellum, immunostained with disease-associated α-syn (5G4), were scanned at 40× magnification using the TissueScope LE120 (Huron, Saint Jacobs, Canada). The putamen and cerebellar white matter were manually outlined and dissected using Adobe Photoshop software. To measure the size of GCIs, a minimum–maximum threshold was established to exclude non-GCI immunoreactivity and overlapping GCIs. Using this threshold on the dissected subregion image, the size of each GCI (in square pixels, px^2^) was quantified using the ‘analyse particles’ tool in ImageJ, after converting the images into binary. The number of GCIs was quantified using the ‘analyse particles’ tool on the image of the dissected subregion with the applied threshold, and the total ‘count’ was recorded. Subsequently, the total GCI ‘count’ was divided by the total area of tissue to calculate GCIs/mm^2^. The density of all types of α-syn pathology was determined using the ‘analyse particles’ tool in ImageJ. The individual morphological variables were pooled and categorized into Aβ-predominant or non-Aβ-predominant groups.

### Double-labelled immunofluorescent staining

Double-labelled immunostaining was conducted on the basal ganglia sections obtained from four cases exhibiting Aβ-predominant ADNC-MSA (MSA 1–4). The primary antibody cocktail targeting Aβ (6F3D) and phosphorylated-α-syn (EP1536Y) was incubated overnight, followed by staining with secondary antibodies labelled with Alexa Fluor 488 and 555, as detailed in Supplementary Table 2. To mitigate autofluorescence, 1% Sudan Black B was introduced. The prepared sections were then mounted using ProLong Gold antifade reagent containing 4′,6-diamidino-2-phenylindole. Image acquisition was executed with a Nikon C2Si+ confocal microscope equipped with a 40× objective lens, and the images were captured using NIS-Elements AR software.

### α-syn seeding amplification assay

α-syn seeding amplification assay was performed to investigate the seeding capacity of the misfolded α-syn present in the putamen. This assay was conducted in three cases of Aβ-predominant ADNC-MSA (MSA 1–3), one case of intermediate form Aβ-predominant ADNC-MSA (MSA 5) and four cases of non-Aβ-predominant ADNC-MSA (MSA 12, 14, 19 and 20), as previously reported.^27,28^

### Genetic analysis

The genotypes for *APOE* and microtubule-associated protein tau (*MAPT*) and the presence of pathogenic mutations in amyloid precursor protein (*APP*) and the presenilin genes (*PSEN1* and *PSEN2*) were examined as previously described.^29^

### ADNC density plot

Both A (Thal Aβ phases) and B (Braak NFT stages) scores in ADNC were plotted for all *APOE* genotype available MSA and non-MSA cases (*n* = 143), and a regression line and 95% confidence intervals were generated using JMP 14.3 software (JMP Statistical Discovery LLC, Cary, NC, USA). The plotted density distribution was represented as the quantile density contour.

### Literature review

We conducted a literature review of Aβ-predominant ADNC-MSA cases using PubMed on 1 November 2023. The search terms included ‘multiple system atrophy’ AND ‘autopsy’; ‘multiple system atrophy’, AND ‘pathology’; ‘multiple system atrophy’ AND ‘Alzheimer’; ‘multiple system atrophy’ AND ‘Alzheimer’s disease’, ‘multiple system atrophy’ AND ‘amyloid’; ‘multiple system atrophy’ AND ‘Aβ’. The inclusion criteria to define Aβ-predominant ANDC-MSA encompassed cases featuring individuals with a Thal phase of 4 or higher and a Braak NFT stage of II or lower, corresponding to National Institute on Aging-Alzheimer’s Association ADNC ABC scores A3B1 and A3B0.

### Statistical analysis

Categorical variables were analysed using Fisher’s exact test, while continuous variables were analysed using the Mann–Whitney *U* test. Propensity score matching was used to compare the frequency of Aβ-predominant ADNC cases and *APOE* ɛ4 carriers between MSA and non-MSA cases. Statistical analyses were conducted using SPSS Statistics (version 23, IBM, Chicago, IL, USA) and JMP 14.3 (JMP Statistical Discovery LLC, Cary, NC, USA). Significance levels were set at *P* < 0.05 utilizing a two-tailed approach.

## Results

### Summary of the demographic and clinical features of the cohort

Table 1 summarizes the clinical, genetic and pathological features of MSA cases, which consisted of 21 consecutive MSA patients, with 15 of them being female. Detailed clinical, genetic, radiological and pathological features were also summarized in Supplementary Table 3. All patients were confirmed to have MSA through autopsy. Among them, four cases (19%; MSA 1–4) exhibited a Thal phase of 5, and one case (MSA 5) was Thal Phase 3, with corresponding Braak stages ranging from 1 to 2. The remaining cases had Thal Phases 0–2 with Braak Stages 0–II. The four cases with Thal Phase 5 had peculiar characteristics as described below; we defined them as ‘Aβ-predominant ADNC-MSA’ and one case of Thal Phase 3 as ‘intermediate form of Aβ-predominant ADNC-MSA’, to distinguish from the 16 cases lacking this feature (non-Aβ MSA). Among the latter MSA cases, one patient (MSA 6) was pure MSA without any other mixed pathologies, while the remaining 15 cases displayed mixed pathology compatible with PART.^10^ All patients received a diagnosis of clinically established or probable MSA,^1^ except for one patient in the non-Aβ-predominant ADNC-MSA (MSA 7), diagnosed with clinically established Parkinson’s disease.^30^ There were no statistically significant differences in age at onset, age at death, disease duration, clinical MSA subtypes (MSA-P or MSA-C) or the presence of cognitive impairment, Parkinsonism, cerebellar signs, autonomic failure or levodopa responsiveness between Aβ and non-Aβ-predominant MSA cases. The frequency of the *APOE* ɛ4 allele was not significantly different between Aβ-predominant ADNC-MSA and non-Aβ MSA cases (3 out of 3 cases versus 5 out of 15 cases, *P* = 0.07). There was no significant difference in the prevalence of carriers with *MAPT* H1/H1 or H2/H2 haplotypes between Aβ-predominant and non-Aβ-predominant patients. None of the Aβ-predominant cases had mutations in *APP*, *PSEN1* or *PSEN2*. Radiologically, the frequency of typical MSA findings,^1^ encompassing atrophy in the putamen, brainstem (including the ‘hot-cross-bun sign’) and cerebellum, did not differ between Aβ-predominant ADNC-MSA and non-Aβ MSA cases. Pathologically, Aβ-predominant ADNC-MSA cases displayed a higher incidence of CAA compared with non-Aβ MSA cases (4 out of 4 cases versus 2 out of 16 cases, *P* = 0.003).

**Table 1:** Summary of the clinical, genetic and pathological findings of the MSA cohort

	Clinical findings				Genetics		Pathological findings	
Case	Sex	Age at death	Disease duration, years	Clinical diagnosis	ApoE	MAPT	ADNC	CAA type*
MSA 1	F	64	6	MSA-P	3/4	H1/H1	A3B1C2 (T5, BrII)	Type 1
MSA 2	F	61	3	MSA-C	4/4	H1/H1	A3B1C2 (T5, BrI)	Type 1
MSA 3	F	74	6	MSA-P	3/4	H1/H1	A3B1C2 (T5, BrII)	Type 1
MSA 4	F	69	6	MSA-P	n.a.	n.a.	A3B1C2 (T5, BrI–II)	Type 1
MSA 5	M	69	12	MSA-C	3/3	H1/H2	A2B1C1 (T3, BrI)	Type 2
MSA 6	F	46	6	MSA-P	3/4	H1/H1	A0B0C0 (T0, Br0)	-
MSA 7	F	46	6	PD	3/3	H1/H1	A0B1C0 (T0, BrI)	-
MSA 8	F	76	6	MSA-P	3/4	H2/H2	A0B1C0 (T0, BrII)	-
MSA 9	M	64	4	MSA	3/3	H1/H1	A0B1C0 (T0, BrII)	-
MSA 10	M	62	10	MSA-C	3/3	H1/H1	A0B1C0 (T0, BrII)	-
MSA 11	M	62	15	MSA-C	3/3	H1/H1	A0B1C0 (T0, BrII)	-
MSA 12	F	61	4	MSA-P	3/3	H1/H1	A0B1C0 (T0, BrI)	-
MSA 13	M	68	5	MSA-P	3/3	H1/H1	A0B1C0 (T0, BrII)	-
MSA 14	F	76	4	MSA-P	3/3	H1/H1	A0B1C0 (T0, BrI)	-
MSA 15	M	73	7	MSA	3/3	H1/H2	A1B1C0 (T1, BrI)	-
MSA 16	F	69	4	MSA-P	3/3	H1/H1	A1B1C0 (T2, BrII)	-
MSA 17	F	68	6	MSA	3/4	H1/H1	A1B1C0 (T1, BrII)	Type 1
MSA 18	F	71	7	MSA-P	2/4	H1/H1	A1B1C1 (T2, BrII)	-
MSA 19	F	64	5	MSA-P	3/3	H1/H1	A1B1C0 (T1, BrI)	-
MSA 20	F	66	8	MSA-P	2/4	H1/H1	A1B1C0 (T1, BrI)	Type 2
MSA 21	F	66	7	MSA-C	n.a.	n.a.	A1B1C1 (T1, BrI)	-

Cases 1–4 represent Aβ-predominant ADNC-MSA cases.

ADNC, Alzheimer’s disease neuropathologic change; APOE, apolipoprotein E: Br, Braak NFT stage; CAA, cerebral amyloid angiopathy; n.a, not available; T, Thal Aβ phase.

*P < 0.05, MSA 1–4 versus MSA 5–21 by Fisher’s exact test.

### Pathological findings of MSA with divergent Aβ phase and NFT stage

**Figure 1:**
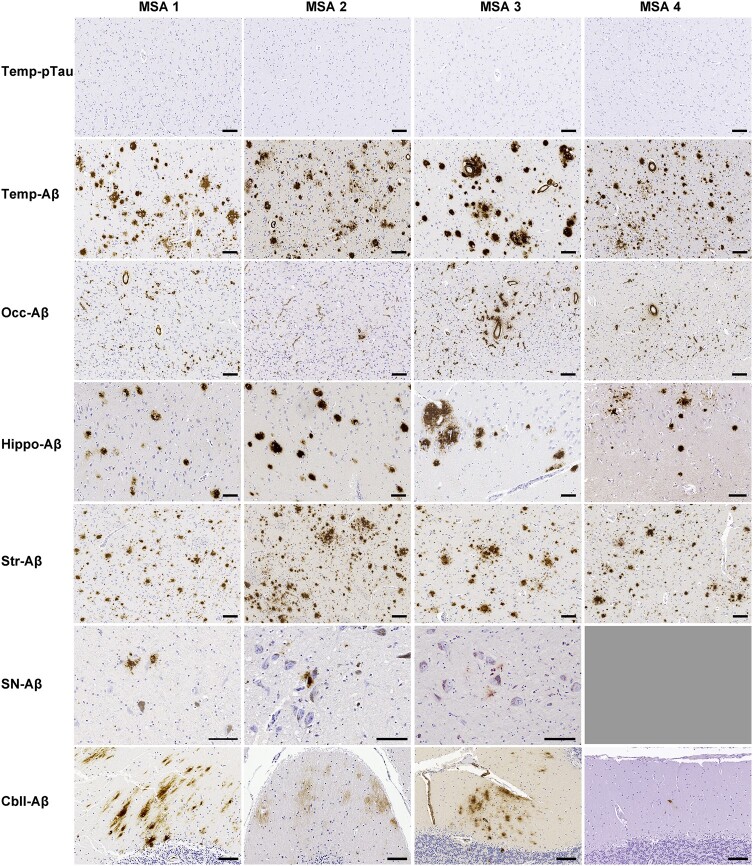
**Representative images of Aβ-predominant Alzheimer’s disease neuropathological changes MSA.** Absence of phosphorylated tau immunoreactivity in the temporal cortex, contrasting with widespread and severe Aβ pathologies throughout the brain. The substantia nigra was not available in MSA 4. Bars represent 100 μm. Cbll, cerebellum; Hippo, hippocampus; Occ, occipital cortex; pTau, phosphorylated tau; SN, substantia nigra; Str, striatum; Temp, temporal cortex.

We identified four cases that fulfilled the criteria for ADNC A3B1, which we refer to Aβ-predominant ADNC-MSA cases. Figure 1 and Table 2 present the pathological features of the four Aβ-predominant MSA cases. All cases exhibited neuronal loss, α-syn-ir NCIs and GCIs in both the striatonigral and olivopontocerebellar systems. These findings were consistent with typical MSA pathology. Only one case (MSA 1) showed a few α-syn-ir NCIs in the hippocampus. Regarding Aβ pathology, diffuse plaques were abundant in the temporal cortex and striatum in all four cases, in the frontal cortex in three out of four cases and in the occipital cortex in two out of four cases. Less amount of cored plaques was observed in the cerebral cortices in all four cases. A defining characteristic of these cases was that tau pathology was minimal in all four. CAA pathology was present with capillary CAA being prominent in all four cases in the cerebral cortices and cerebellum, particularly in the occipital cortex. All four cases showed relatively uniform pathology, with many diffuse plaques in the cerebral cortices and basal ganglia, and prominent capillary CAA in the cerebral cortices with the most severe in the occipital cortex in three carries of *APOE* ɛ4 allele (MSA 4 was unavailable for genetic testing). Vascular pathologies, except for CAA, as well as Lewy-type and TDP-43 pathologies, were absent in all Aβ-predominant ADNC-MSA cases (Table 2). Given that the occurrence of mixed pathology involving Lewy-type, TDP-43 and vascular pathologies in MSA is only 5–8% in a large cohort^17^ and our Aβ-predominant ADNC-MSA cases did not contain any of these pathologies, we concentrated on the assessment of ADNC pathology in the subsequent analyses.

**Table 2:** Pathological features of MSA cases with Aβ-predominant ADNC

	MSA 1	MSA 2	MSA 3	MSA 4
**Lewy pathology**	None	None	None	None
**TDP-43 pathology**	None	None	None	None
**MSA pathology**				
**Putamen**				
Neuronal loss	4	0	4	4
α-Synuclein-positive NCI/GCI	4/4	1/2	4/4	4/4
**Substantia nigra**				
Neuronal loss	3	2	3	n.a.
α-Synuclein-positive NCI/GCI	3/4	2/2	2/3	n.a.
**Pontine base**				
Neuronal loss	2	4	1	n.a.
α-Synuclein-positive NCI	3/4	3/4	2/3	n.a.
**Inferior olivary nuclei**				
Neuronal loss	2	4	0	n.a.
α-Synuclein-positive NCI	2/1	3/1	1/1	n.a.
**Cerebellum**				
Neuronal loss (Purkinje cells)	2	3	2	2
α-Synuclein-positive NCI/GCI	0/4	0/4	0/3	0/2
**Hippocampus**				
Neuronal loss	0	0	0	0
α-Synuclein-positive NCI/GCI	2/2	0/1	0/1	0/1
**Aβ pathology (6F3D)**				
**Frontal cortex**				
Diffuse plaque/cored plaque	4/2	4/2	4/2	2/1
CAA capillary/non-capillary	3/0	3/0	4/3	3/3
**Temporal cortex**				
Diffuse plaque/cored plaque	4/2	4/2	4/2	4/2
CAA capillary/non-capillary	3/0	3/0	3/0	3/0
**Occipital cortex**				
Diffuse plaque/cored plaque	1/1	4/1	3/2	1/0
CAA capillary/non-capillary	3/4	4/4	4/4	4/4
**Hippocampus**				
Diffuse plaque/cored plaque	3/0	3/0	3/0	1/0
CAA capillary/non-capillary	2/3	1/0	2/0	0/0
**Striatum**				
Diffuse plaque/cored plaque	4/0	4/0	4/1	4/0
CAA capillary/non-capillary	0/0	0/0	1/0	2/0
**Midbrain**				
Diffuse plaque/cored plaque	2/0	1/0	1/0	n.a.
CAA capillary/non-capillary	1/0	0/0	0/0	n.a.
**Cerebellum**				
Diffuse plaque/cored plaque	3/0	2/0	3/0	1/0
CAA capillary/non-capillary	3/1	3/3	3/2	3/1

Aβ, amyloid-beta; CAA, cerebral amyloid angiopathy; GCI, glial cytoplasmic inclusion; NCI, neuronal cytoplasmic inclusion; NFT, neurofibrillary tangle.

Next, we compared the Aβ pathology in Aβ-predominant ADNC-MSA with Alzheimer’s disease cases without α-syn, TDP-43 or vascular pathologies, using antibodies for various Aβ peptides in the temporal cortex and striatum (summarized in Supplementary Fig. 1 and Table 3). Aβ-predominant ADNC-MSA cases had lower Braak NFT and CERAD stages compared with Alzheimer’s disease cases with similar Thal Aβ-phase (*P* < 0.001 and *P* < 0.01, respectively). In the temporal cortex and striatum, Aβ-predominant ADNC-MSA cases exhibited fewer cored plaques with pan-Aβ (6F/3D), Aβ_40_, Aβ_42_, Aβ_43_ and Aβ_Np3E_ than Alzheimer’s disease cases (*P* < 0.05). Furthermore, Aβ-predominant ADNC-MSA cases showed fewer Aβ_40_- and Aβ_43_-ir diffuse plaques in the striatum than Alzheimer’s disease cases (*P* < 0.05 and *P* < 0.01, respectively).

**Table 3:** Aβ-peptide molecular signatures between MSA cases with Aβ-predominant ADNC and Alzheimer’s disease cases

	MSA 1	MSA 2	MSA 3	MSA 4	AD1	AD2	AD3	AD4	*P*-value
**Thal Aβ phase**	5	5	5	5	4	5	5	5	n.s.
**Braak NFT stage**	II	I	II	I–II	VI	VI	VI	VI	**<0.001**
**CERAD**	2	2	2	2	3	3	3	3	**<0.01**
**Aβ immunoreactivity**									
**Temporal cortex**									
**Pan-Aβ (6F3D)**									
Diffuse plaque	4	4	4	4	4	4	4	4	n.s.
Cored plaque	2	2	2	2	4	3	3	3	**<0.05**
**Aβ_40_**									
Diffuse plaque	3	2	4	3	3	4	4	3	n.s.
Cored plaque	2	0	1	1	3	3	3	3	**<0.05**
**Aβ_42_**									
Diffuse plaque	4	4	4	4	4	4	4	4	n.s.
Cored plaque	2	2	2	2	4	4	3	3	**<0.05**
**Aβ_43_**									
Diffuse plaque	4	4	4	4	4	4	4	4	n.s.
Cored plaque	2	1	2	2	4	4	3	3	**<0.05**
**Aβ_Np3E_**									
Diffuse plaque	4	4	4	4	4	4	4	4	n.s.
Cored plaque	2	2	2	2	4	3	3	3	**<0.05**
**Aβ_pSer8_**									
Diffuse plaque	3	3	3	3	4	4	3	2	n.s.
Cored plaque	2	1	2	1	3	2	2	2	n.s.
**Striatum**									
**Pan-Aβ (6F3D)**									
Diffuse plaque	4	4	4	4	4	4	4	4	n.s.
Cored plaque	0	0	1	0	2	3	2	1	**<0.05**
**Aβ_40_**									
Diffuse plaque	0	0	1	0	2	3	3	2	**<0.05**
Cored plaque	0	0	0	0	2	3	2	0	**<0.05**
**Aβ_42_**									
Diffuse plaque	4	4	4	4	4	4	4	4	n.s.
Cored plaque	0	0	1	0	2	3	2	1	**<0.05**
**Aβ_43_**									
Diffuse plaque	3	3	3	3	4	4	4	4	**<0.01**
Cored plaque	0	0	0	0	2	3	2	0	**<0.05**
**Aβ_Np3E_**									
Diffuse plaque	4	4	4	4	4	3	4	4	n.s.
Cored plaque	0	0	0	0	2	3	2	1	**<0.05**
**Aβ_pSer8_**									
Diffuse plaque	3	2	3	1	3	3	4	1	n.s.
Cored plaque	0	0	1	0	2	3	0	0	n.s.

Mann–Whitney *U* test is applied for statistics. The values highlighted in bold indicate statistical significance.

Aβ_Np3E_, pyroglutamate Aβ at the third glutamic acid; Aβ_pSer8_, phosphorylated-Aβ at the eighth serine; n.s., not significant.

### Double-labelled immunofluorescent staining

Given the identification of both Aβ plaques and α-syn-ir GCIs in the striatum, further double-labelled immunofluorescent staining was performed. It is noteworthy that Aβ plaques, α-syn-ir GCIs and neurites did not co-localize in GCIs in Aβ-predominant ADNC-MSA; however, due to the diffuse fine granular deposition pattern of Aβ in the neuropil, a few α-syn dots in the neuropil did co-exist in the same locations (Supplementary Fig. 2).

### Morphometry of α-syn-ir GCIs

**Figure 2:**
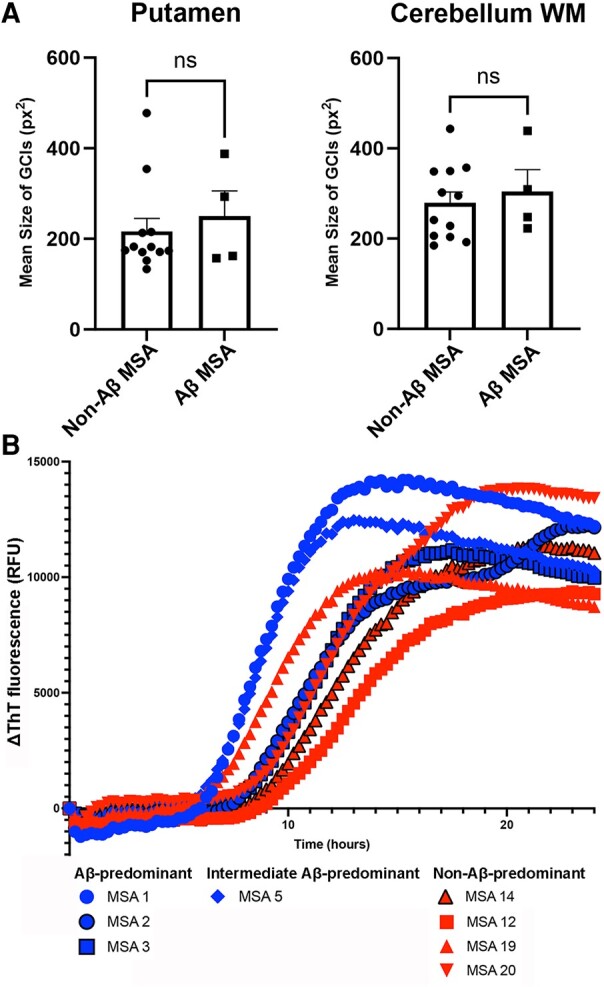
**GCIs morphometry and α-syn seeding amplification assay.** (**A**) The mean size of α-syn-ir GCIs is similar between Aβ-predominant ADNC-MSA and non-Aβ-predominant ADNC-MSA cases in the putamen and cerebellum white matter (*n* = 4 versus 12). Mann–Whitney *U* test is applied for the statistics. (**B**) α-syn seeding amplification assay demonstrates that two cases (MSA 1, Aβ-predominant ADNC-MSA; MSA 5, intermediate form Aβ-predominant) exhibit distinctive seeding kinetics from others (2 Aβ-predominant ADNC-MSA and 4 non-Aβ-predominant ADNC-MSA). ns, not significant; RFU, relative fluorescent unit; WM, white matter.

The mean size of GCIs, the number of GCIs/mm^2^ and the area density of all types of α-syn pathology in the putamen and cerebellum white matter were similar between Aβ-predominant ADNC-MSA cases and non-Aβ MSA cases (Fig. 2A and Supplementary Fig. 3; *n* = 4 versus *n* = 12).

### α-syn seeding amplification assay

α-syn seeding amplification assay was performed in eight cases, including three Aβ-predominant ADNC-MSA (MSA 1–3), one intermediate form Aβ-predominant ADNC-MSA (MSA 5) and four non-Aβ-MSA cases (MSA 12, 14, 19 and 20). Among these, two cases exhibited distinctive seeding curves (Fig. 2B). Both cases had Aβ pathology in the putamen, with one being Aβ-predominant ADNC-MSA (MSA 1) and the other intermediate form Aβ-predominant ADNC-MSA case (MSA 5). In contrast, two other Aβ-predominant ADNC-MSA cases (MSA 2 and 3) and four cases lacking Aβ pathology in the putamen displayed comparable seeding kinetics (Fig. 2B).

### Impact of *APOE* ɛ4 allele on Aβ-predominant ADNC pathology

**Figure 3:**
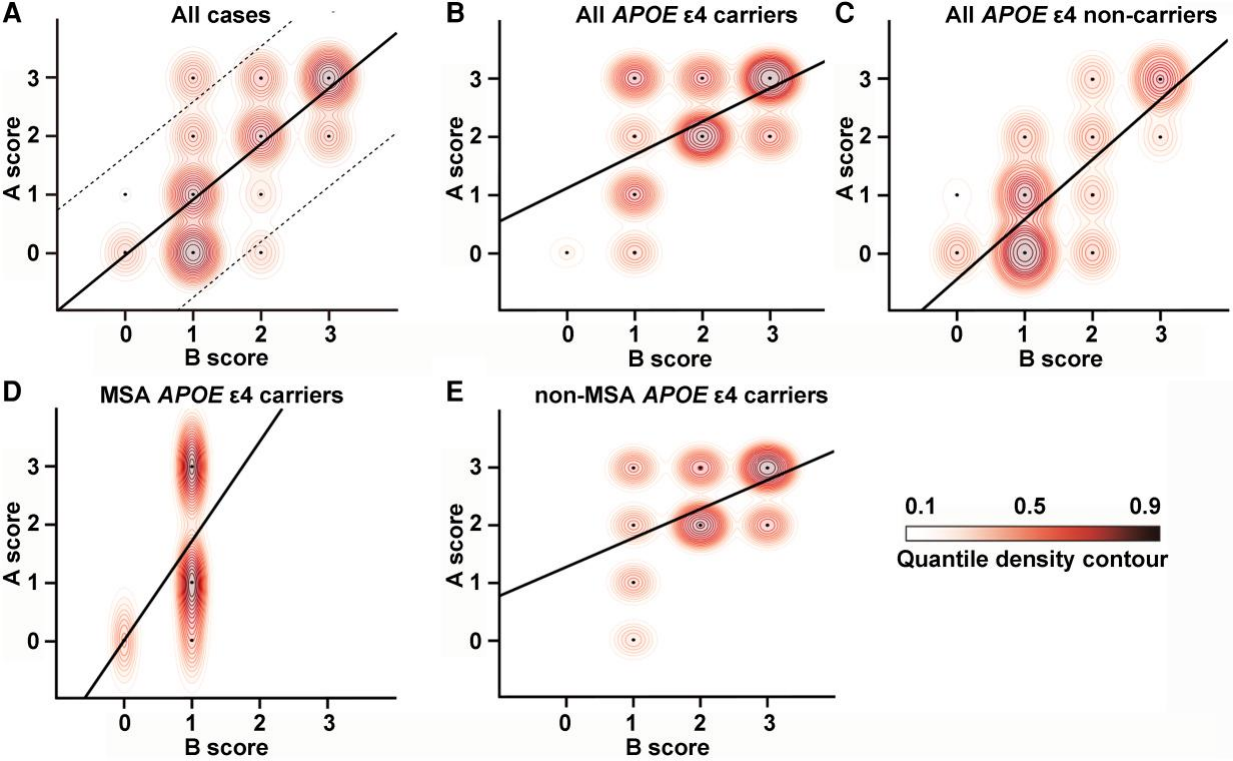
**National Institute on Aging-Alzheimer’s Association ADNC score plot.** ADNC A and B scores in all cases (**A**, *n* = 143), all *APOE* ɛ4 carriers (**B**, *n* = 61), all *APOE* ɛ4 non-carriers (**C**, *n* = 82), MSA *APOE* ɛ4 carriers (**D**, *n* = 8) and non-MSA *APOE* ɛ4 carriers (**E**, *n* = 53). The regression line (**A–E**) and the upper and lower 95% confidence intervals (**A**) are graphically represented.

To explore whether the discrepancy between extensive Aβ but minimal tau pathology is associated with the presence of *APOE* ɛ4 allele, we further examined Alzheimer’s disease neuropathologic change in 124 consecutive various non-MSA-type neurodegenerative diseases and 19 MSA cases for which *APOE* genotypes were available. Among these cases, 53 (42.7%) were *APOE* ɛ4 carriers. The frequency of *APOE* ɛ4 carriers did not differ between MSA and non-MSA cases (8/19 versus 53/124, *P* = 1). In the non-MSA cohort, only four cases (3.2%) that were all *APOE* ɛ4 carriers exhibited ADNC score A3B1 (see Supplemental Table 1). The frequency of Aβ-predominant ADNC cases was significantly higher in MSA than in non-MSA cases (3/19 versus 4/124, *P* < 0.05). However, when age and sex were matched using propensity scores, there were no statistical differences (*P* = 0.6). In Fig. 3A, ADNC A and B scores, the regression line and 95% confidence intervals are plotted in MSA and non-MSA cases with available *APOE* genotypes (*n* = 143), revealing a strong correlation between ADNC A and B scores (*r* = 0.71, *P* < 0.001), with ADNC A3B1 above the upper limit of the 95% confidence interval. Figure 3B and C shows ADNC A and B scores in all *APOE* ɛ4 carriers (*n* = 61) and all *APOE* ɛ4 non-carriers (*n* = 82), respectively, suggesting the presence of *APOE* ɛ4 increases the baseline of the A score. Figure 3D and E depicts ADNC A and B scores in MSA *APOE* ɛ4 carriers (*n* = 8) and non-MSA *APOE* ɛ4 carriers (*n* = 53), respectively, indicating that the regression line is steeper in MSA than in non-MSA cases, which is supporting the observation of the discrepancy between A and B scores in our MSA cohort.

### Literature review

Our literature review identified three Aβ-predominant ADNC-MSA cases out of a total of 38 cases reported.^5,31,32^ These cases are summarized in Table 4. The prevalence of Aβ variant MSA cases was found to be 3.8–9.1% in each cohort, with a total pooled prevalence of 7.9% (excluding a single case report). While this prevalence appears lower compared with our cohort, it did not show a statistically significant difference (*P* = 0.22). The median age at death was 68, and there was no statistical significance in comparison with our Aβ variant cases. Disease duration was reported for two patients (21 and 12 years), while one patient was not described. Cognitive status was only reported in two patients who showed no impairment, but their cognition was not formally evaluated. *APOE* genotype was reported for only one patient, which was as ɛ3/ɛ4.

**Table 4:** Previously reported MSA cases with Aβ-predominant ADNC

Authors	Coughlin *et al*.^31^	Koga *et al*.^32^	Robinson *et al*.^5^
Year	2022	2020	2018
Country	USA	USA	USA
Number of cases	1	1	1
ADNC	A3B1C1 (T5, BrII)	A3B1Cn.d. (T4, BrII)	A3B0-1Cn.d. (T4–5, Br 0–II)
Pathological MSA types	Mixed	Mixed (mixed pathology with LBD)	n.d.
Clinical MSA subtypes	MSA-P	MSA-C	n.d.
Age at death (years)	66	70	n.d.
Disease duration (years)	21	12	n.d.
Sex	F	M	n.d.
Percentages in the cohort	100% (1/1)	9.1% (1/11)	3.8% (1/26)
Cognitive impairment	n.d.	-	n.d.
Cognitive test	n.e.	n.e.	n.d.
Initial symptoms	Parkinsonism + autonomic	Gait ataxia	n.d.
Parkinsonism	+	+	n.d.
Cerebellar symptoms	−	+	n.d.
Autonomic failure	+	+	n.d.
L-DOPA efficacy	−	n.d.	n.d.
ApoE4 genotypes	n.d.	ɛ3ɛ4	n.d.
MRI findings	Hot cross ban +, marked atrophy in the pons and cerebellum	n.d.	n.d.

ADNC, Alzheimer’s disease neuropathologic change; APOE, apolipoprotein E: Br, Braak NFT stage; LBD, Lewy body disease; n.d, not described; n.e., not examined; T, Thal Aβ phase.

## Discussion

In this study, we present four cases of MSA with a discrepancy between a high Thal phase of Aβ deposition and a low Braak NFT stage. This discrepancy is unusual and unexpected even given the patients’ *APOE* genotype status and shorter disease duration than if they had pure Alzheimer’s disease without MSA. These cases showed only mild or no cognitive impairment, and the clinical and radiological features were not significantly different from cases without Aβ pathology.

ADNC is scored by staging schemes based on neuroanatomical hierarchy. Thal Aβ phase progresses from the neocortex through the hippocampus, basal ganglia/diencephalon, brainstem and cerebellum.^8^ Braak NFT staging begins in the entorhinal and limbic areas and progresses to neocortical regions.^7^ The prevalence of Alzheimer’s disease-related pathology increases with advancing age.^33,34^ NFTs are found in over 80% of individuals in their 60s,^33,34^ while Aβ plaques are observed in 30% of individuals in their 60s and 50–60% of individuals in their 80s.^33,34^ Mixed pathologies are less frequent and typically mild in MSA patients.^5,6,12-14,16,17^ Based on these reports and our study, we consider four types of MSA based on ADNC-type mixed pathology: pure MSA, MSA with PART, MSA with typical ADNC where Braak NFT stages and Thal Aβ phases increase in parallel as reported in single case reports^35,36^ and larger MSA cohorts,^5,12-17^ and finally the Aβ-predominant ADNC-MSA as highlighted in this study.

Out of 21 cases in our MSA cohort, four cases (19%) exhibited the Aβ variant. The literature review revealed only three cases of Aβ-predominant ADNC-MSA (Table 4).^5,31,32^ The prevalence of Aβ-predominant ADNC-MSA cases in our consecutive case collection is unexpectedly high (19%) compared with other studies (7.9%). Jellinger^12,13^ and other groups^37-39^ have reported Aβ alone co-pathology in MSA cases, but these reports did not specify the Thal phase; therefore, the Aβ-predominant ADNC-MSA might be higher in some cohorts. Although several studies evaluated a larger number of MSA cases, they either did not report ADNC^38,40,41^ or reported only the mean or median values of Thal phases and Braak NFT stages for the pooled cohort^13-15,42^ or did not report the ADNC score suggesting the discrepancy of extent and severity between Aβ and tau pathology has been under-recognized. A recent study reported Thal phases and Braak NFT stages^17^; however, a discrepancy between Thal phases and Braak stages in a degree reported here was not mentioned even in carriers of *APOE* ɛ4 allele. In a forensic cohort of young subjects (aged 30–65 years), mild Aβ pathology without any tau deposition was observed only in 23/431 subjects (5.3%), and the majority of these subjects carried the *APOE* ɛ4 allele.^43^ This finding supports the notion that the presence of Aβ plaques in the cortex precedes NFT pathology and could be linked to genetic predisposition (e.g. *APOE* ɛ4). However, that forensic cohort did not report cases with Aβ pathology involving subcortical areas, brainstem and cerebellum with only minimal or no tau pathology. Cases with Aβ-predominant ADNC-MSA in our cohort carried the *APOE* ɛ4 allele. In the literature, only pooled data are available in connection to *APOE* genotypes, and a single case with *APOE* genotype (ɛ3/ɛ4) and Aβ pathology was reported.^32^ In our cohort, the *MAPT* haplotypes showed no association with Aβ-predominant ADNC-MSA cases, and the presence of pathogenic Alzheimer’s disease mutations in *APP*, *PSEN1* or *PSEN2* was excluded. The *APOE* ɛ4 allele is strongly associated with the typical constellation ADNC (i.e. plaques and tangles),^5,44^ and both Aβ and tau pathologies typically advance concurrently.^45-47^ Our investigation reveals that a significant proportion of *APOE* ɛ4 carriers, encompassing five out of eight cases within the MSA cohort and 50 of 53 cases within the non-MSA cohort, did not manifest the discrepancy between Aβ and tau pathologies. Consequently, the *APOE* ɛ4 alleles alone does not seem to be the driving force for the discrepancy between high Aβ Thal phases and low NFT Braak stages. Therefore, further genetic factors or ethnicity might contribute to variations in the reported frequencies of mixed pathologies. Furthermore, the reason for the high prevalence of Aβ-predominant ADNC-MSA in our cohort remains unclear since the age of our MSA patients is comparable with MSA cases reported from around the world. Unfortunately, larger cohorts, which report ADNC co-pathologies in MSA cases, do not specifically describe Aβ Thal phases and Braak NFT stages for each case, making it difficult to explore the frequency of Aβ-predominant ADNC-MSA cases. We believe that raising attention of this condition will facilitate collaborative efforts to explore whether there are peculiar aspects or genetic constellations that we might have missed due to the limited number of cases examined.

Importantly, the amyloid cascade hypothesis postulates that Aβ induces tau pathology^48^; however, in Aβ-predominant ADNC cases, this pattern cannot be recognized. Indeed, Aβ-predominant ADNC cases are associated only with mild cognitive alterations, if any, supporting the notion that tau pathology is more associated with cognitive decline.^49^ We are aware that the high Thal phase with low Braak NFT stage is not specific to MSA, as similar variants have been reported in other diseases, although they may have been under-recognized. Terry *et al*.^50^ described ‘plaque-only dementia’ in 18 out of 60 (45%) Alzheimer’s disease cases in 1987, and later, they reported its association with Lewy pathology.^51^ A similar pathology has also been observed in 5 out of 29 (17.2%) Down syndrome cases.^52^ However, it is worth noting that these studies did not apply modern immunohistochemistry techniques including staining for Aβ, and ‘plaque-only dementia’ meant to represent cases with neuritic plaques without tangles in the cortex. Indeed, this is completely different from the concept of Aβ-predominant ADNC-MSA where the focus is on the distribution of Aβ deposits involving the striatum, brainstem and cerebellum as well. With the advancements in modern immunohistochemistry, cases with a predominant presence of Aβ plaques compared with tau pathology have rarely been reported, but their recognition remains limited. In population-representative cohorts in the UK, the prevalence of high Thal with low Braak stage co-pathologies ranged from 1.1% (six cases of Thal 4–5 with Braak NFT II in 186 cases)^53^ to 1.9% (two cases of Thal 4–5 with Braak NFT I–II in 106 patients).^54^ Additionally, this type of co-pathology has been observed in 2.6% of cases (five cases of Thal 4–5 with Braak NFT 0–II in 192 Alzheimer’s disease patients) in a multi-centre study in the USA.^55^ In progressive supranuclear palsy cohorts, the prevalence of moderate to high Thal phase with low Braak stage co-pathology ranged from 3.4% (1/29 patients, Thal 3 with Braak I)^56^ to 4.9% (4/81 patients, two cases of Thal 5 with Braak II and two cases of Thal 3 with Braak I).^57^ In a community-based study from Austria (VITA study), a similar discrepancy was not found.^4^ A recent large international cohort evaluating the frequency of limbic-predominant age-related TDP-43 encephalopathy neuropathological change (LATE-NC) revealed that among cases without LATE-NC, 41 out of 2334 (1.7%) cases had Thal Phase 4 or 5 associated with Braak NFT Stages 0–II.^11^

In Lewy body disease, the prevalence of high Thal phase and low Braak stage co-pathology has been reported to range from 3.2% (21/652 cases, Thal 4–5 with Braak Stage 0–II) to 4.5% (1/22 cases, Thal 4 with Braak II).^58,59^ Kotzbauer *et al*.^60^ reported that 19 of 32 patients (59%) with Parkinson’s disease dementia exhibited Aβ deposition (Braak amyloid Stages B and C) but little to moderate tau deposition (Braak NFT Stages 0–IV), although Thal phases were not reported in their cohort. Interestingly, Aβ accumulation in synucleinopathies appears to be more frequent than in tauopathies, suggesting a potential synergistic relationship between the pathological aggregation of α-syn and Aβ.^61-63^ The accumulation of α-syn may further disrupt protein homoeostasis and contribute to the pathological accumulation of Aβ.^61,62^ Understanding the mechanisms underlying this mixed pathology may shed light on specific interactions with α-syn and common mechanisms involving other proteins that contribute to neurodegeneration. Recent studies have identified distinct strains of Aβ,^19,64^ and cryo-electron microscopy analysis has revealed polymorphisms in Aβ fibrils.^65,66^ It is plausible that potential unknown interactions between α-syn and Aβ, or unidentified features of α-syn or Aβ, may contribute to the development of Aβ variant pathology.

In our cases with Aβ-predominant ADNC-MSA, morphologically diffuse plaques predominated, with fewer cored plaques compared with Alzheimer’s disease. The median age at death in Aβ-predominant ADNC-MSA cases was 66.5 years old, which is younger than typical Alzheimer’s disease cases where dementia usually develops after age 65.^44^ Diffuse plaques precede the development of cored plaques.^29,30,61,62^ Additionally, the prevalence of NFT pathology increases with age.^33,34,67,68^ Therefore, we cannot exclude the possibility that patients with Aβ variant of MSA have not lived long enough to develop significant NFT pathology.

Cerebral cortex and striatal Aβ deposition are important pathological findings closely associated with the development of dementia in individuals with Lewy body disease.^69,70^ We have recently reported that the molecular signature of Aβ peptides differs among various neurodegenerative diseases.^18,19^ Therefore, we conducted further analyses of the Aβ peptides in Aβ variant of MSA, which demonstrated significantly fewer cored plaques in the temporal cortex and striatum than Alzheimer’s disease cases using pan-Aβ, Aβ_40_, Aβ_42_, Aβ_43_ and Aβ_Np3E_ antibodies. Additionally, we observed fewer diffuse plaques in the striatum compared with Alzheimer’s disease in Aβ_40_ and Aβ_43_ immunoreactivity. Notably, the severity of Aβ_pSer8_-ir diffuse and cored plaques in Aβ variant of MSA was similar to Alzheimer’s disease. Regarding the biochemical maturation process of Aβ in Alzheimer’s disease and/or Down syndrome, Aβ_42_ and Aβ_43_ are the first Aβ species to accumulate in the human brain.^52,71,72^ Aβ_40_ is detected subsequently, followed by Aβ_Np3E_ and/or Aβ_Np11E_,^52,71,72^ and finally, Aβ_pSer8_ accumulates.^67,72^ In Alzheimer’s disease patients and transgenic mouse brains, Aβ_43_ has been found in diffuse and cored plaques, with Aβ_43_ having the strongest propensity to aggregate, followed by Aβ_42_ and Aβ_40_.^73^ Aβ_pSer8_ is mainly restricted to symptomatic Alzheimer’s disease.^67^ Fewer cored plaques in cases of Aβ variant of MSA than Alzheimer’s disease suggest that Aβ plaques in this variant are premature. On the other hand, the Aβ_pSer8_ burden was similar to Alzheimer’s disease, indicating that Aβ plaques in this variant still contain the peptide thought to be toxic at a biochemical level.^67^ The molecular signature of Aβ peptides might be influenced by the local α-syn accumulation contributing to the development of the pathology of the Aβ variant and cognitive impairment.

Prominent capillary CAA in the cerebral cortices, particularly in the occipital cortex, characterizes the advanced Thal phase of Aβ variant of MSA. This distribution and severity of Aβ plaques and CAA pattern align with Type 3, as reported by Allen *et al*.^69^ Previous studies have indicated that capillary CAA is strongly associated with the *APOE* ɛ4 allele and higher Thal phase.^53,74^ In line with these findings, three out of four genotyped Aβ-predominant ADNC-MSA cases (one case lacked genetic testing) carried the *APOE* ɛ4 allele. The prevalence of *APOE* ɛ4 carriers in our total MSA cohort was 42.1% (8/19), which appears higher than previous reports of 22%.^5,75^ The *APOE* ɛ4 allele is strongly associated with the typical constellation ADNC (i.e. plaques and tangles),^5,44^ and CAA is also an independent contributor to cognitive impairment.^76^ Although it is plausible that the *APOE* ɛ4 allele may influence the distribution and severity of Aβ plaques and CAA pathology in Aβ-predominant ADNC-MSA, however, we demonstrated that *APOE* ɛ4 alone might not account for the discrepancy between Aβ Thal phases and Braak NFT stages.

In our study, Aβ-predominant ADNC-MSA cases exhibited only mild or no cognitive impairment. Previous reports have suggested that cognitive impairment in MSA is associated with the load of α-syn-ir NCIs in the hippocampus.^14,41,42^ Clinical features of frontotemporal lobar degeneration with abundant α-syn-ir NCIs in the frontotemporal cortices and limbic systems are referred to as ‘FTLD-synuclein’.^77,78^ MSA patients with severe α-syn-ir NCIs in the hippocampus have been referred to as ‘hippocampal MSA’.^42^ Therefore, it is conceivable that a particular subset of individuals with MSA may demonstrate increased susceptibility to α-syn-ir NCIs in the limbic system. However, only one case (MSA 1) of Aβ-predominant ADNC-MSA showed mild α-syn-ir NCIs in the hippocampus, while all others did not. Therefore, the mild cognitive impairment in our cases appears to be independent of the hippocampal α-syn-ir NCIs.

In two cases of MSA where both α-syn and Aβ were present in the putamen, distinct kinetic curves were observed compared with other MSA cases with and without Aβ in α-syn seeding amplification assay. Given the reported cross-seeding effects between α-syn and Aβ,^61,79-82^ we cannot exclude the possibility that the presence of Aβ could influence the unique α-syn kinetic curves. Although we did not observe any differences in the morphometry or co-localization between Aβ and α-syn in GCIs, the co-presence of fine granular Aβ deposits α-syn dots in the neuropil support the notion that these two proteins have the opportunity to interact. These aspects merit further studies when such cases will be identified in other cohorts.

**Figure 4:**
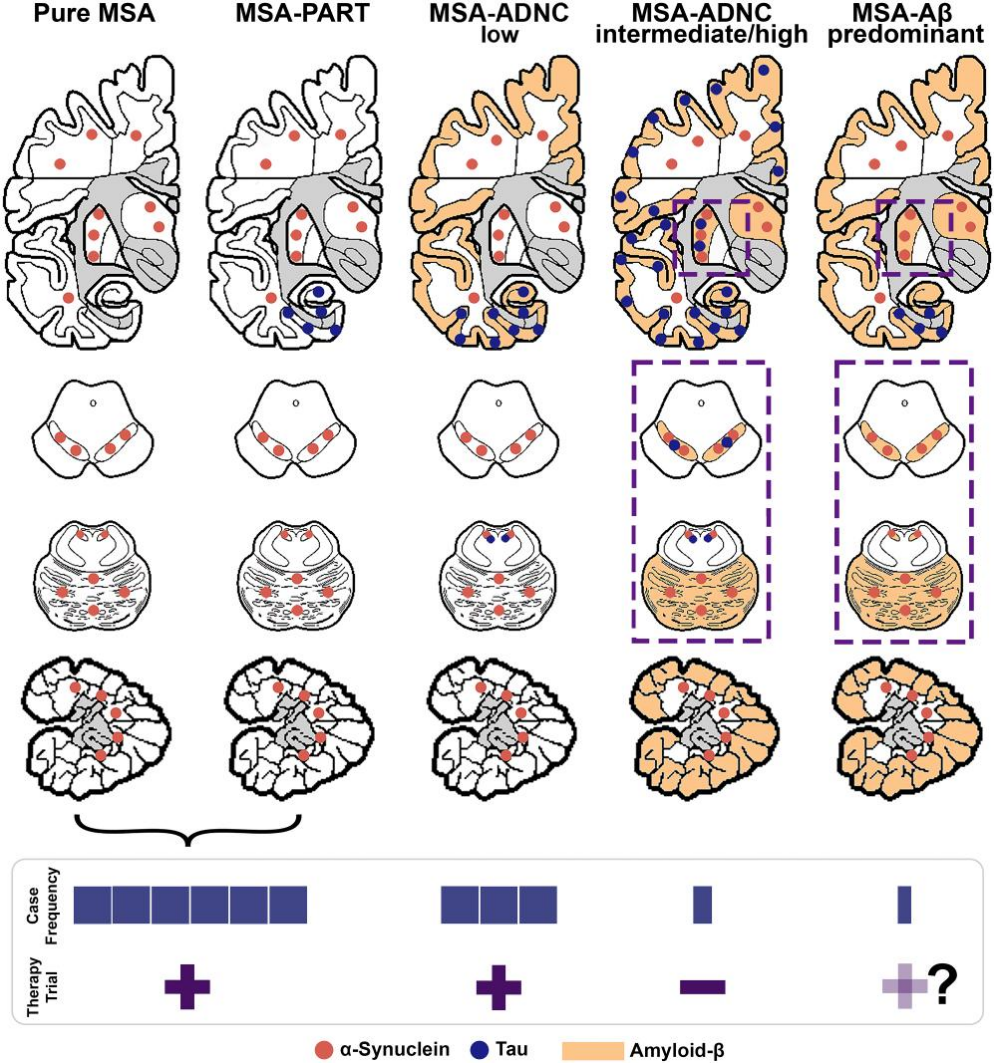
**Spectrum of mixed pathology of ADNC in MSA.** Five constellations are shown: the most frequent is pure MSA pathology or MSA pathology associated with NFTs in the entorhinal cortex and hippocampus as in PART followed by low or less frequently intermediate and high levels of ADNC. The bar graph shows the case frequency of each subgroup. The dashed-line square represents the anatomical meeting points of multiple proteins. Regarding the consideration of using α-syn-targeted therapy in patients with high-level ADNC would largely be excluded due to the severe cognitive decline (indicated by a dash in the row indicating α-syn therapy trial). In contrast, cases of MSA with PART, MSA with low-level ADNC and Aβ-predominant ADNC-MSA groups might be included since the clinical phenotype is not significantly different (indicated by a ‘+’ in the row indicating α-syn therapy trial). Importantly, in Aβ-predominant ADNC-MSA cases, the possibility of an interaction between α-syn and Aβ is the highest, and therefore, this might have an effect on the outcome of the α-syn therapy trial and might justify the consideration of combined therapies separately targeting the two proteinopathies.

In conclusion, this study has unveiled the neuropathological features of Aβ-predominant ADNC-MSA, which were previously unappreciated, raising awareness of this variant. The limitation of our study is the relatively small number of patients included. Therefore, the exact frequency and specific clinical signature of this variant cannot be determined. Further larger studies are needed to determine whether there is a yet unidentified genetic aspect or other reason for the clustering detected in our cohort. These cases originated from a racially very diverse population in Toronto Canada so a genetic founder effect accounting for the proportion of such cases in our sample is highly unlikely. The stratification of this variant may be crucial for clinicians to provide precise treatments for subtypes of MSA. We propose a novel two-tiered approach to the interpretation of mixed pathologies to address the questions of whether (i) the additional misfolded protein contributes to or alters the clinical phenotype and (ii) there is an anatomical overlap between the misfolded protein deposits that allow direct interaction. Indeed, the Aβ-predominant ADNC-MSA variant is the most prone to fulfil the second premise, which theoretically could lead to an altered response to α-syn-directed therapies. Furthermore, disease-modifying therapies targeting Aβ have been rapidly evolving^83,84^ and could be considered for combined therapies in such cases. Furthermore, in the Aβ-predominant ADNC-MSA variant, tau pathology, which is the major reason for accelerated cognitive impairment in cases with typical ADNC,^34,49,55^ does not have an impact on the clinical progression. Since biomarker studies are entering clinical practice, these cases will be recognized, and the clinicians have to be informed that the prognosis is not necessarily different than in pure MSA cases and also that the effects of potential α-syn-based therapies might be influenced by the co-presence of amyloid in regions where α-syn also aggregates. Therefore, we believe that informing the clinicians about this phenotype is important. If one envisions the future of personalized medicine, the identification of variants that can be diagnosed with *in vivo* tau, Aβ and synuclein-based biomarkers is of high importance. Our study contributes to the understanding of the full spectrum of mixed pathology variants in MSA (summarized in Fig. 4).

## Supplementary Material

fcae141_Supplementary_Data

## Data Availability

The data that support the findings of this study are available from the corresponding author upon reasonable request.
