# Supplementary material for: Multiple system atrophy with amyloid-β-predominant Alzheimer’s disease neuropathologic change
Source: Brain Commun. 2024 Apr 17;6(3):fcae141. doi: 10.1093/braincomms/fcae141 (PMC11073746; doi:10.1093/braincomms/fcae141)
Supplement: fcae141_Supplementary_Data [file fcae141_supplementary_data.pdf]

## Supplementary material for the article

## Supplementary method

### Morphometry of α-syn-ir GCIs

The morphological variables, encompassing size, density, number of GCIs, and density of all αSyn pathology, were assessed and analyzed in accordance with established protocols.[1] In summary, sections of the basal ganglia and cerebellum, immunostained with disease-associated α-synuclein (α-syn) antibody (clone 5G4),[2] underwent scanning at 40X magnification utilizing the TissueScopeTM LE120 and TissueSnapTM (Huron, Saint Jacobs, Canada). Images of the putamen and cerebellar white matter, both standardized in size, were captured at 2.06X magnification and subsequently cropped to 31416 x 10800 pixels using Adobe Photoshop. The putamen and cerebellar white matter were outlined and dissected with the lasso tool.

To precisely capture GCIs throughout an entire anatomical subregion using a semi-automated approach, we established a size threshold. Employing Adobe Photoshop, we randomly selected and individually dissected 100 αSyn-positive GCIs with visible nuclei onto a new canvas of the same size at 300 dpi using the magic wand tool. Subsequently, the image underwent flattening, brightness and contrast adjustment to 100%, and conversion to grayscale. Size threshold values were determined by loading the image onto Image J (NIH, Bethesda, MD), converting it to binary, and measuring each inclusion in square pixels ($px^2$) using the Analyze Particle function. Results were exported to Excel (Microsoft, Redmond, WA), where the minimum and maximum GCI areas from the randomly sampled 100 inclusions were collected for each image. The minimum threshold (i.e., the smallest GCI size with a visible nucleus) aimed to eliminate non-specific background staining, dot- and thread-like inclusions. The maximum threshold (i.e., the largest GCI size with a visible nucleus) was set to exclude GCIs in close proximity (e.g., multiple inclusions touching each other), particularly in regions with high GCI density.

For semi-automated size measurement of each GCI in regions of interest, α-syn

immunoreactivity was initially dissected using the color range selection tool in each pre-processed image. Subsequently, these selections were pasted onto a new canvas of the same size at 300 dpi and saved. The image with all αSyn immunoreactivity in the putamen and cerebellar white matter (WM) was loaded into Image J and measured as described earlier, but with the minimum and maximum thresholds applied to capture only GCIs and avoid background noise or overlapping inclusions. The area ($px^2$) of each αSyn GCI falling between the minimum and maximum thresholds in the putamen or cerebellar WM was recorded for each case.

We assessed the density of α-syn pathology, encompassing various morphologies (i.e., neuronal cytoplasmic and nuclear, neuritic, and glial), as detected by the 5G4 α-syn antibody specific to disease-associated α-syn. We quantified the density (i.e., total area) of all types of α-syn immunoreactivity across the entire tissue area in both the putamen and cerebellar WM for each case. Initially, to gauge the total tissue area, pre-processed images were imported into Photoshop, and tissues were outlined using the object selection tool to create an overlaying shape. Subsequently, the image was saved and transferred to Image J, where it was converted to binary, and the area of the shape was measured in square pixels ($px^2$). For the assessment of the density of all α-syn pathology, the previously generated image with all α-syn immunoreactivity was loaded onto Image J, and the total area of all α-syn immunoreactivity was quantified using the analyze particles tool. The resulting value, representing the total area of all types of disease-associated αSyn immunoreactivity, was then divided by the total tissue area to yield the density of all α-syn pathology as a percentage of the total area.

Subsequently, we directed our attention specifically to the hallmark lesion and quantified the density of GCIs in both the putamen and cerebellar WM. The previously generated image containing all α-syn immunoreactivity was loaded into Image J, and utilizing the established threshold, we measured the total area occupied by α-syn-positive GCIs using the analyze particles tool. The resulting value, representing the total area of α-syn-positive GCIs, was then divided by the total tissue area to calculate the density of GCIs as a percentage.

Moving on to assess the number of α-syn immunoreactive GCIs in brain region, the

previously created image with all α-syn immunoreactivity was loaded into Image J, and with the pre-established threshold applied, we measured the total number of α-syn-positive GCIs using the analyze particles tool. Given the variability in the area of total tissue evaluated among cases and regions, we normalized the number of GCIs by dividing it by the total tissue area, converting square pixels ($px^2$) to square millimeters ($mm^2$), thus generating the number of GCIs per unit area in $mm^2$.

**Supplementary references**


1. Kim A, Yoshida K, Kovacs GG, Forrest SL. Computer-based evaluation of α-synuclein pathology in multiple system atrophy as a novel tool to recognize disease-subtypes. Under revision.
2. Kovacs GG, Wagner U, Dumont B, et al. An antibody with high reactivity for disease-associated alpha-synuclein reveals extensive brain pathology. *Acta Neuropathol.* 2012;124(1):37-50.

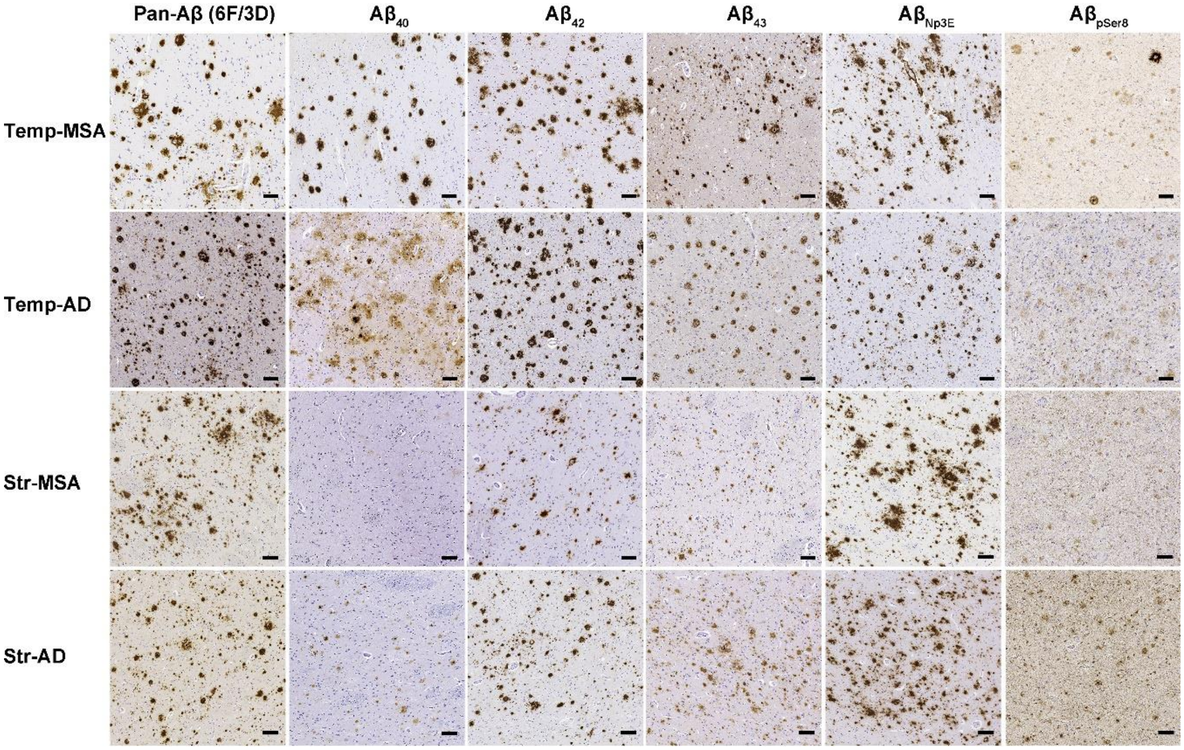


**Supplementary Fig. 1 Representative immunohistochemical images of various Aβ peptides in Aβ-predominant ADNC MSA and Alzheimer's disease (AD) cases.**

Temporal cortex (Temp) and striatum (Str) are shown. Aβ, amyloid-beta; $A\beta_{Np3E}$, pyroglutamate Aβ at 3rd glutamic acid; $A\beta_{pSer8}$, phosphorylated Aβ at 8th serine. Bars represent 100 μm.

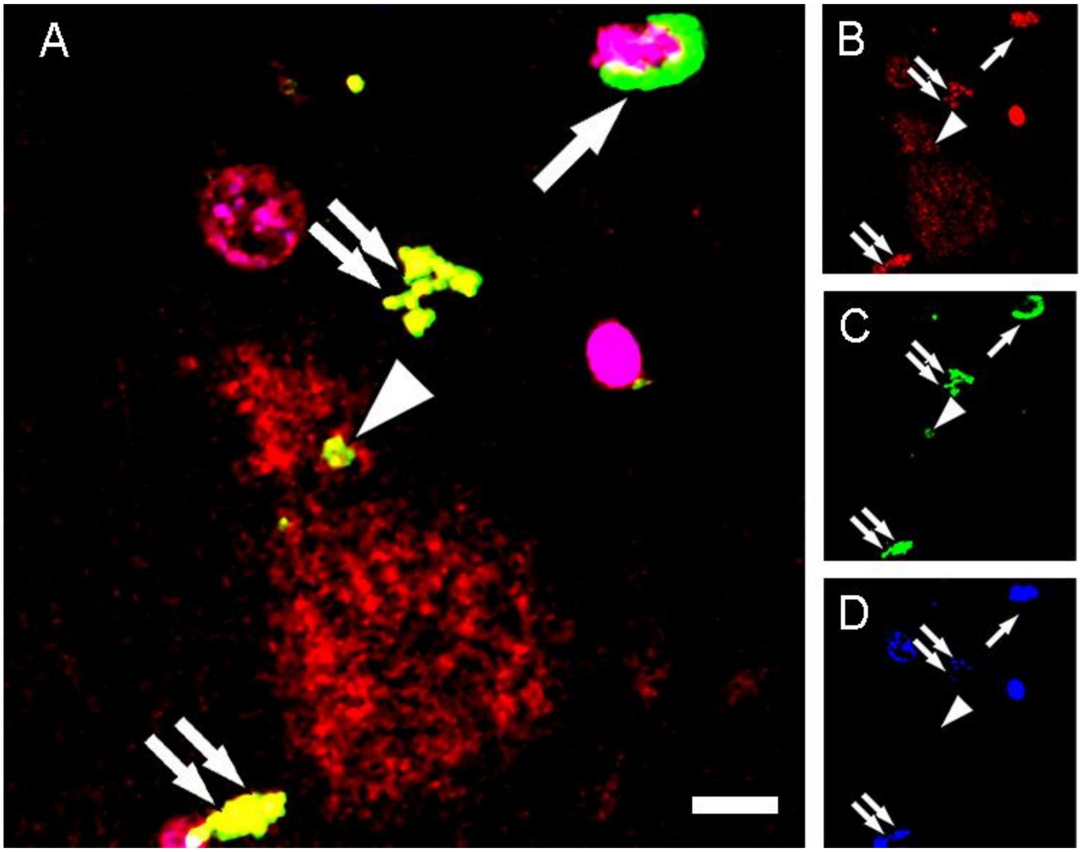


**Supplementary Fig. 2 Double-labeled immunofluorescence in the striatum.**

Merged image (A), diffuse plaques labeled by Aβ antibody (B), glial cytoplasmic inclusions (arrow) and neurites (arrowheads) labeled by phosphorylated-α-synuclein antibody (C), and nuclei and autofluorescence (double arrow) labeled by 4′,6-diamidino-2-phenylindole (D) in the striatum in Aβ-predominant ADNC MSA. While Aβ does not directly co-localize with α-synuclein in glial cytoplasmic inclusions, a few α-synuclein dots co-exist (arrowheads) in the neuropil with the diffuse fine granular deposition pattern of Aβ in the neuropil. Each image set represents confocal images taken from the same field of view showing Aβ immunostaining (B), phosphorylated-α-synuclein immunostaining (C), and 4′,6-diamidino-2-phenylindole (D). Note: Due to the fact that 5G4 α-synuclein is not compatible regarding clonality with the Aβ antibody for double labelling, we used a monoclonal rabbit phosphorylated-α-synuclein antibody that shows much less α-syn pathology. Bar represents 5 μm.

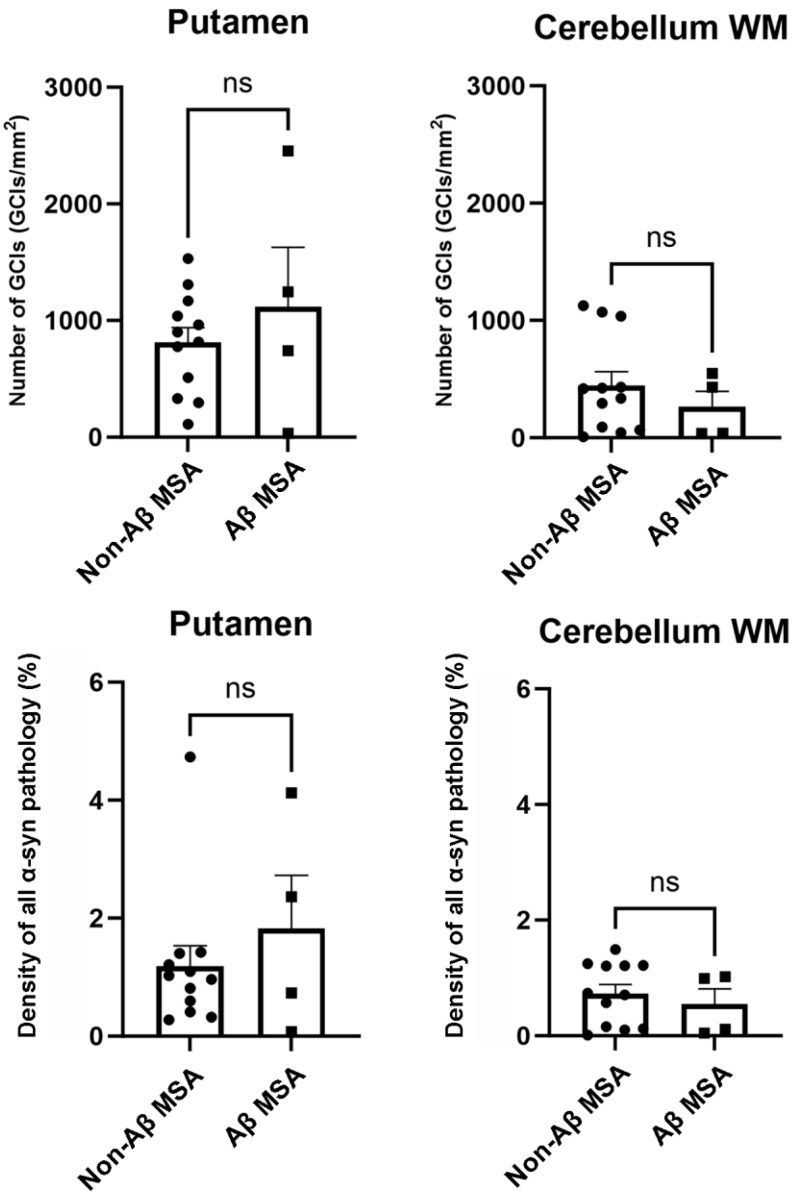


**Supplementary Fig. 3 Glial cytoplasmic inclusions (GCIs) number and density of all α-synuclein pathology in the putamen and cerebellum white matter (WM)**

The number of α-synuclein (α-syn) immunoreactive GCIs and the density of all α-syn pathology are not different between Aβ-predominant ADNC MSA (n = 4) and non-Aβ-predominant ADNC MSA cases (n = 12) in the putamen and cerebellum WM. Mann-Whiteney U test is applied for the statistics. ns, not significant.

**Supplementary Table 1 Summary of cases except for MSA.**

| **Diagnosis** | **n** | ***APOE* ε4 carrier, n (%)** | **NIA-AA ADNC A3B0—1, n (%)** |
|---|---|---|---|
| Lewy body disease | 52 | 32 (61.5%) | 1 (1.9%) |
| Progressive supranuclear palsy | 35 | 13 (37.1%) | 2 (5.7%) |
| Alzheimer's disease | 14 | 4 (28.6%) | 0 |
| Frontotemporal lobar degeneration | 10 | 1 (10%) | 1 (10%) |
| Corticobasal degeneration | 4 | 0 | 0 |
| Control | 3 | 1 (33.3%) | 0 |
| Down syndrome | 3 | 2 (66.7%) | 0 |
| Chronic traumatic encephalopathy | 2 | 0 | 0 |
| Others | 1 | 0 | 0 |

APOE, apolipoprotein.

**Supplementary Table 2 Summary of antibodies and pretreatments used in this study.**

| Antibody | Source | Clone | Dilution | 1st Antigen retrieval | 2nd Antigen retrieval |
|---|---|---|---|---|---|
| Pan-Aβ | Dako | Mo, mc, 6F/3D | 1:50 (IHC), 1:20 (IF) | 80% FA 1h | None |
| $A\beta_{40}$ | BioLegend | Rb, mc, QA18A67 | 1:3000 (IHC) | 70% FA 10 min | None |
| $A\beta_{42}$ | BioLegend | Rb, mc, 1-11-13 | 1:500 (IHC) | 70% FA 10min | None |
| $A\beta_{43}$ | IBL | Rb, polyclonal (18583) | 1:100 (IHC) | 88% FA 5 min | None |
| $A\beta_{Np3E}$ | BioLegend | Mo, mc, 337.48 | 1:800 (IHC) | Heat[a] | 88% FA 3 min |
| $A\beta_{pSer8}$ | Sigma-Aldrich | Mo, mc, 1E4E11 | 1:200 (IHC) | Heat[a] | 88% FA 3 min |
| α-syn | Analytikjena | Mo, mc, 5G4 | 1:4000 (IHC) | Heat[a] | 80% FA 5 min |
| Phosphorylated-α-syn (Ser129) | Abcam | Rb, mc, EP1536Y | 1:500 (IF) | 80% FA 1h | None |
| Phosphorylated-tau (Ser202, Thr205) | ThermoFischer | Mo, mc, AT8 | 1:1000 (IHC) | Heat[a] | None |
| Phosphorylated-TDP-43 | CosmoBio | Mo, mc, 11-9 | 1:2000 (IHC) | Heat[a] | 80% FA 1 min |
| Alexa Fluor 488 | Thermo Fisher Scientific | Rb, mc, A11034 | 1:500 (IF) | Not applicable | Not applicable |
| Alexa Fluor 555 | Thermo Fisher Scientific | Mo, mc, A31570 | 1:500 (IF) | Not applicable | Not applicable |

[a] Performed using Dako PT Link with low pH solution. Aβ, amyloid-beta; AβNp3E, pyroglutamate Aβ at 3rd glutamic acid; AβpSer8, phosphorylated Aβ at 8th serine; FA, formic acid; IF, immunofluorescence; IHC; immunohistochemistry; mc, monoclonal; Mo, mouse; Rb, rabbit.

**Supplementary Table 3 Detailed clinical, genetic, radiological, and pathological findings**

| Clinical findings | | | | | | | | | | | | Genetics | | | | Radiological findings | Pathological findings | | |
|---|---|---|---|---|---|---|---|---|---|---|---|---|---|---|---|---|---|---|---|
| Case | Sex | Age at death | Disease duration, years | Clinical diagnosis | Initial symptoms | Cognitive impairment | Cognitive test | Parkinsonism | Cerebellar signs | Autonomic failure | L-DOPA response | ApoE 4 | APP | PSEN | MAPT | MRI | ADNC | CAA type | Other mixed-pathology |
| MSA 1 | F | 64 | 6 | MSA-P | Urinary incontinence | + (short-term memory loss) | MoCA 26/30 | + | - | + | Initially responsive | 3/4 | - | - | H1/H1 | Normal | A3B1C2 (T5, BrII) | Type I | - |
| MSA 2 | F | 61 | 3 | MSA-C | Postural instability | + (short-term memory loss) | MoCA 24/30 | - | + | + | Not tried | 4/4 | - | - | H1/H1 | Hot cross ban sign | A3B1C2 (T5, BrI) | Type I | - |
| MSA 3 | F | 74 | 6 | MSA-P | Gait difficulty | - | MoCA 25/30 | + | - | + | Minimal response | 3/4 | - | - | H1/H1 | Putaminal atrophy | A3B1C2 (T5, BrII) | Type I | - |
| MSA 4 | F | 69 | 6 | MSA-P | Bradykinesia | n.e.* | n.e. | + | - | + | Initially responsive | n.a. | n.a. | n.a. | n.a. | Putaminal atrophy | A3B1C2 (T5, BrI-II) | Type I | - |
| MSA 5 | M | 69 | 12 | MSA-C | Gait difficulty | + problems with short-term memory, attention, and verval fluency) | MoCA 27/30, PD-CRS 64-81 | - | + | + | Not tried | 3/3 | - | - | H1/H2 | Atrophy in the cerebellum, pons, putamen | A2B1C1 (T3, BrI) | Type 2 | - |
| MSA 6 | F | 46 | 6 | MSA-P | Drooling | - | n.a. | + | - | + | + | 3/4 | n.e. | n.e. | H1/H1 | n.a. | A0B0C0 (T0, Br0) | - | - |
| MSA 7 | F | 46 | 6 | PD | Parkinsonism | - | - | + | n.a. | n.a. | Initially responsive | 3/3 | n.e. | n.e. | H1/H1 | n.a. | A0B1C0 (T0,BrI) | - | - |

| | | | | | | | | | | | | | | | | | | | |
|---|---|---|---|---|---|---|---|---|---|---|---|---|---|---|---|---|---|---|---|
| MSA 8 | F | 76 | 6 | MSA-P | Gait and writing difficulties | n.a. | n.a. | + | + | + | n.a. | 3/4 | n.e. | n.e. | H2/H2 | n.a. | A0B1C0 (T0, BrII) | - | - |
| MSA 9 | M | 64 | 4 | MSA | Balance issues | + | MMSE 30/30/TorCA 269 | + | + | + | n.a. | 3/3 | n.e. | n.e. | H1/H1 | atrophy in the cerebellum, pons, putamen | A0B1C0 (T0, BrII) | - | AGD |
| MSA 10 | M | 62 | 10 | MSA-C | Gait and writing difficulties | + | MoCA 24/30 | + | + | + | Initially responsive | 3/3 | n.e. | n.e. | H1/H1 | Atrophy in the cereberum, cerebellum, and putamen. Hot cross bun sign, | A0B1C0 (T0, BrII) | - | ARTAG |
| MSA 11 | M | 62 | 15 | MSA-C | n.a. | n.a. | n.a. | n.a. | n.a. | + | n.a. | 3/3 | n.e. | n.e. | H1/H1 | n.a. | A0B1C0 (T0, BrII) | - | - |
| MSA 12 | F | 61 | 4 | MSA-P | Slowness of movement | - | n.a. | + | - | + | - | 3/3 | n.e. | n.e. | H1/H1 | Mild atrophy in the putamen and coreberum | A0B1C0 (T0, BrI) | - | - |
| MSA 13 | M | 68 | 5 | MSA-P | Generalized weakness and voice changes | - | MoCA 27/30 | + | - | + | - | 3/3 | n.e. | n.e. | H1/H1 | Putaminal atrophy | A0B1C0 (T0, BrII) | - | AGD |
| MSA 14 | F | 76 | 4 | MSA-P | Tremor and slowness of movements | - | Not found | + | - | + | - | 3/3 | n.e. | n.e. | H1/H1 | Mild atrophy in the putamen and coreberum | A0B1C0 (T0, BrI) | - | - |

| | | | | | | | | | | | | | | | | | | | |
|---|---|---|---|---|---|---|---|---|---|---|---|---|---|---|---|---|---|---|---|
| MSA 15 | M | 73 | 7 | MSA | Shoulder Pain | n.a. | n.a. | + | + | + | n.a. | 3/3 | n.e. | n.e. | H1/H2 | Putaminal atrophy | A1B1C0 (T1, BrI) | - | Cerebral hemorrhages in the pons and thalamus |
| MSA 16 | F | 69 | 4 | MSA-P | Gait difficulty | - | n.a. | + | - | + | + | 3/3 | n.e. | n.e. | H1/H1 | Putaminal atrophy | A1B1C0 (T2, BrII) | - | ARTAG, LATE |
| MSA 17 | F | 68 | 6 | MSA | Tremor | n.a. | n.a. | + | n.a. | n.a. | n.a. | 3/4 | n.e. | n.e. | H1/H1 | n.a. | A1B1C0 (T1, BrII) | Type 1 | ARTAG |
| MSA 18 | F | 71 | 7 | MSA-P | Tremor | - | n.a. | + | - | + | + | 2/4 | n.e. | n.e. | H1/H1 | Normal | A1B1C1 (T2, BrII) | - | - |
| MSA 19 | F | 64 | 5 | MSA-P | Gait difficulty | - | n.a. | + | + | + | - | 3/3 | n.e. | n.e. | H1/H1 | Mild cerebellar atrophy | A1B1C0 (T1, BrI) | - | - |
| MSA 20 | F | 66 | 8 | MSA-P | Urinary urgency and erectile dysfunction | - | MoCA 29/30 | + | + | + | - | 2/4 | n.e. | n.e. | H1/H1 | Mild atrophy in the putamen and parietal cortex | A1B1C0 (T1, BrI) | Type 2 | - |
| MSA 21 | F | 66 | 7 | MSA-C | Balance problems, and speech difficulty | - | Not found | - | + | + | Not tried | n.a. | n.a. | n.a. | n.a. | Atrophy in the cerebellum.Hot-cross bun sign | A1B1C1 (T1, BrI) | - | - |

*Treatment resistant severe depression

Aβ, amyloid-beta; ADNC, Alzheimer's disease neuropathologic change; AF, autonomic failure; AGD, argyrophilic grain disease; APOE, apolipoprotein E: APP, amyloid precursor protein; ARTAG, aging-related tau astrogliopathy; Br, Braak NFT stage; CAA, cerebral amyloid angiopathy; CI, cognitive impairment; CS, cerebellar signs; CT, cognitive tests; DD, disease duration; Dx, clinical diagnosis; LATE, limbic-predominant age-related TDP-43 encephalopathy; MoCA, Montreal cognitive assessment; MMSE, mini-mental state examination; n.a, not available; n.e. not examined; P, parkinsonism; PD-CRS, Parkinson's disease-cognitive rating scale; PSEN, presenilin; S, sex; T, Thal Aβ phase; TorCA, Toronto cognitive assessment.
